# Heterotypic interactions regulate cell shape and density during color pattern formation in zebrafish

**DOI:** 10.1242/bio.022251

**Published:** 2016-10-14

**Authors:** Prateek Mahalwar, Ajeet Pratap Singh, Andrey Fadeev, Christiane Nüsslein-Volhard, Uwe Irion

**Affiliations:** Max Planck Institute for Developmental Biology, Spemannstrasse 35, Tübingen 72076, Germany

**Keywords:** Pigment pattern formation, Cell-cell interactions, Gap junctions, Xanthophores, Iridophores, Melanophores, Zebrafish

## Abstract

The conspicuous striped coloration of zebrafish is produced by cell-cell interactions among three different types of chromatophores: black melanophores, orange/yellow xanthophores and silvery/blue iridophores. During color pattern formation xanthophores undergo dramatic cell shape transitions and acquire different densities, leading to compact and orange xanthophores at high density in the light stripes, and stellate, faintly pigmented xanthophores at low density in the dark stripes. Here, we investigate the mechanistic basis of these cell behaviors *in vivo*, and show that local, heterotypic interactions with dense iridophores regulate xanthophore cell shape transition and density. Genetic analysis reveals a cell-autonomous requirement of gap junctions composed of Cx41.8 and Cx39.4 in xanthophores for their iridophore-dependent cell shape transition and increase in density in light-stripe regions. Initial melanophore-xanthophore interactions are independent of these gap junctions; however, subsequently they are also required to induce the acquisition of stellate shapes in xanthophores of the dark stripes. In summary, we conclude that, whereas homotypic interactions regulate xanthophore coverage in the skin, their cell shape transitions and density is regulated by gap junction-mediated, heterotypic interactions with iridophores and melanophores.

## Abstract (precis)

**Summary:** The conspicuous pigmentation pattern of zebrafish is produced by three kinds of interacting pigment cells. Here we address the cellular consequences of these interactions in wild-type fish and mutants with altered pigment patterns.

## INTRODUCTION

**Fig. 1.**
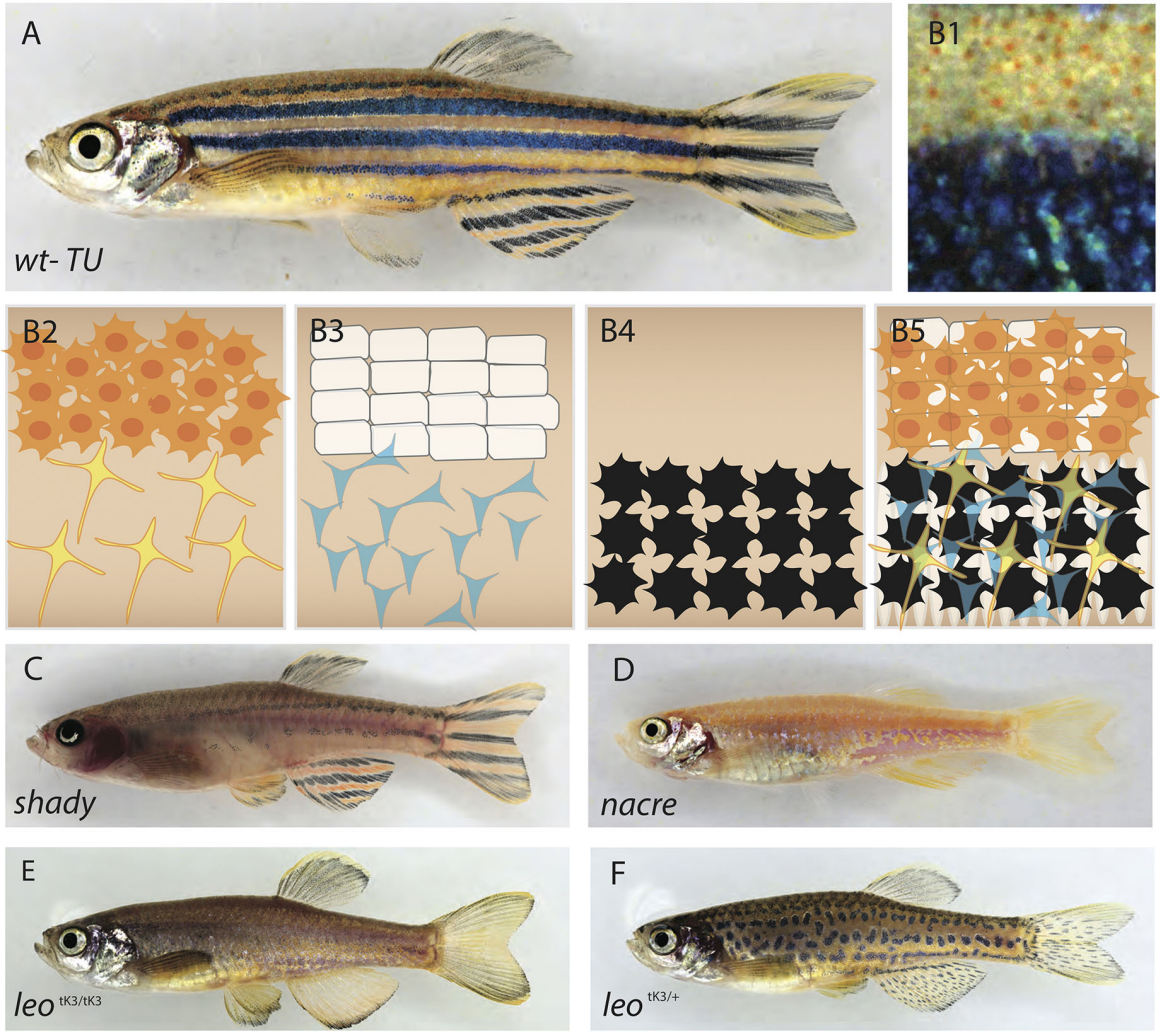
**Pigment cell organization in wild-type and mutant zebrafish.** (A) Wild-type zebrafish, and (B1) close up view of the stripe pattern showing light and dark stripe regions. (B2-5) Schematic organization of pigment cells: (B2) xanthophores are compact and densely packed in the light stripe, loose and stellate in the dark stripe; (B3) iridophore layer beneath xanthophores – epithelial-like packing of silvery iridophores in the light stripe, loose and blue in the dark stripe; (B4) melanophores are only present in the dark stripe region; (B5) precise superimposition of all the three cell types results in golden light stripes and blue/black dark stripes. (C) *shady* lack most iridophores. (D) *nacre* lacks all the neural crest-derived melanophores. (E) Homozygous *leopard^tK3/tK3^*. (F) Heterozygous *leopard ^tK3/+^.*

The striped coloration of adult zebrafish has emerged as a model system to study pattern formation by cell-cell interaction *in vivo* (Irion et al., 2016; Kelsh, 2004; Parichy and Spiewak, 2015; Singh and Nüsslein-Volhard, 2015; Watanabe and Kondo, 2015). The pattern of longitudinal dark and light stripes on the flank of the fish is composed of black melanophores, orange/yellow xanthophores and silvery or bluish iridophores. This color pattern is produced during a phase called metamorphosis, by the precise arrangement and superimposition of all three cell types in the skin of the fish (Hirata et al., 2003, 2005; Parichy et al., 2009). In the dark stripes, melanophores are covered by loose blue iridophores and a net of highly branched (stellate) and faintly colored xanthophores; whereas the light stripes are composed of an epithelial-like sheet of dense iridophores covered by a compact net of orange xanthophores (Fig. 1A) (Mahalwar et al., 2014; Singh et al., 2016, 2014). Homotypic interactions between cells of the same type determine their collective migration and spacing during pattern formation (Walderich et al., 2016). Although it is clear that heterotypic interactions among all three types of pigment cells are necessary to generate the striped pattern (Frohnhöfer et al., 2013; Irion et al., 2014a; Singh et al., 2015), the outcome of these heterotypic interactions at the cellular level is not understood. In mutants where one type of pigment cells is absent [for example *nacre/mitfa* lacking melanophores (Lister et al., 1999), *pfeffer/csf1ra* lacking xanthophores (Parichy et al., 2000b) or *shady/ltk* lacking iridophores (Fadeev et al., 2016; Lopes et al., 2008)], only remnants of the normal stripe pattern are formed by the remaining two types of cells (see examples in Fig. 1). Together with data from ablation experiments (Nakamasu et al., 2009; Yamaguchi et al., 2007), this indicates that a number of heterotypic interactions among the different types of pigment cells are essential for the generation of the pattern (Frohnhöfer et al., 2013; Maderspacher and Nüsslein-Volhard, 2003). At the molecular level a few components mediating these heterotypic interactions have been identified so far, including gap junctions and ion channels (Irion et al., 2014a; Iwashita et al., 2006; Watanabe et al., 2006). Gap junctions are membrane channels allowing the communication between neighboring cells, and we have previously shown that two different subunits of gap junctions, Cx41.8 and Cx39.4 encoded by the *leopard* (*leo*) (Watanabe et al., 2006) and *luchs* (*luc*) (Irion et al., 2014a) genes, respectively, are required in melanophores and xanthophores, but not in iridophores for normal pattern formation. In both *leo* and *luc* loss-of-function mutants the dark melanophore stripes are dissolved into spots, and the light stripe areas are expanded. Dominant hypermorphic alleles of both *leo* and *luc* exist, and they lead to meandering melanophore patterns or even spots in heterozygous fish. Double mutants for *leo* and *luc* loss-of-function alleles display a very severe phenotype, the pattern is completely dissolved with single melanophores scattered on a uniform light sheet of epithelial-like dense iridophores covered by a net of xanthophores. Mutants homozygous for the strongest of the dominant *leo* alleles, *leo^tK3^*, show the same strong phenotype, arguing that both connexins can form heteromeric and homomeric gap junctions (Irion et al., 2014a; Maderspacher and Nüsslein-Volhard, 2003), which was confirmed by *in vitro* studies (Watanabe et al., 2015). This suggests that the communication between xanthophores and melanophores via heteromeric gap junctions provides signals to the dense iridophores to induce the transition into the loose shape required for dark stripe formation.

Key features of the stripe patterning process are the acquisition of precise cell shapes, as well as the correct cell density and the appropriate coloration. Xanthophores acquire their shape, density and color in a context-dependent manner, in the light stripe areas they are present at high density as compact and bright orange cells, whereas they are stellate, faintly pigmented and at lower density in the dark-stripe regions. To investigate the cellular and molecular basis of these cell behaviors, here we use fluorescently labeled xanthophores combined with long-term *in vivo* imaging in various mutants that affect pigment cell development and their interactions. We observe that heterotypic interactions with iridophores and melanophores regulate context-dependent changes in cell shape and density of xanthophores. We show that dense iridophores are required to instruct xanthophores to increase in density and adopt a compact shape. The cellular interactions leading to these behaviors depend on gap junctions formed by *leo* and *luc*. Further, we show that melanophore-xanthophore interactions can be divided into two phases: an initial phase, independent of *leo/luc* gap junctions, and a later phase, during which these junctions are essential. Our results emphasize the importance of an *in vivo* model to study heterotypic cell-cell interactions and their consequences for cell proliferation and behavior, as well as the suitability of zebrafish to investigate the role of gap junctions *in vivo*.

## RESULTS

### Xanthophore density is reduced in mutants lacking iridophores, and in mutants lacking functional gap junctions

**Fig. 2.**
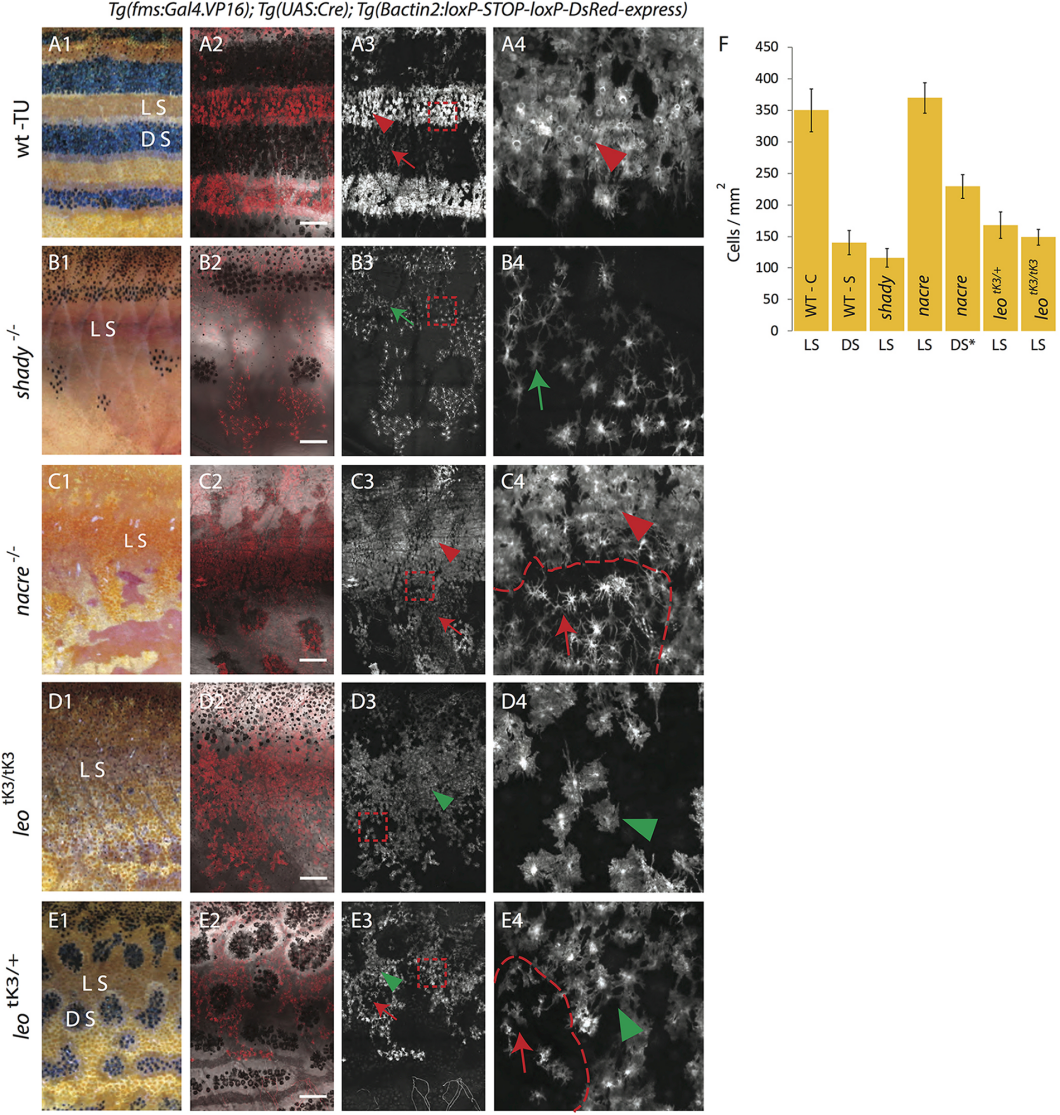
**Density and organization of xanthophores in various pigment cell mutants.** DsRed-positive xanthophores labeled with *Tg*(*fms:Gal4.VP16*); *Tg*(*UAS:Cre*); *Tg*(*βactin2:loxP-STOP-loxP-DsRed-express*) in wild type and mutants. Due to variegated expression of the transgenes not all xanthophores are labelled. (A1-4) Wild type. (A1) Pigmentation pattern in adult wild-type fish (TU), LS, light stripe; DS, dark stripe. (A2) compact xanthophores (arrowhead) in the light stripe and loose xanthophores (arrow) the in dark stripe areas. (A3) A magnified image of the light stripe region in wild-type fish (red dotted box in A3) shows the high density of compact xanthophores (red arrowhead). (B1-4) Homozygous *shady* mutants lacking iridophores. (B1,B2) Overview showing the residual melanophore pattern in the mutant. In (B2,B3) the uniform organization of xanthophores (green arrow) is visible. (B4) Magnified image (red dotted box in B3) shows lower density and different morphology of xanthophores (green arrow). (C1-4) Homozygous *nacre* mutants lacking melanophores. (C1,C2) Overview showing irregular areas of epithelial-like dense iridophores; LS, light stripe. In (C3) the compact (arrowhead) and loose (arrow) shape of xanthophores is visible. (C4) Magnified image (red dotted box in C3) shows high (arrowhead) and low (arrow) density of xanthophores at a light stripe border area (dotted red line). (D1-4) Homozygous *leo^tK3tK3^* mutant. (D1,D2) Overview showing that all three pigment cell types are present. In (D3,D4) the low density and uniform distribution of xanthophores (green arrowhead) is visible; (D4) magnified image (red dotted box in D3) shows uniform cell shape and distribution of xanthophores (green arrowhead). (E1-4) Heterozygous *leo^tK3^* mutants. (E1,E2) Overview indicating the light stripe (LS) and dark stripe (DS) areas. In (E3) the low density but non-uniform distribution of xanthophores in light (green arrow head) and dark (red arrow) stripe regions is visible. (E4) Magnified image (red dotted box in E3) showing stellate (arrow) and compact (green arrowhead) xanthophores with lower density at a melanophore spot boundary (dotted red line). Scale bars: 500 µm. (F) Graph showing the density (number of xanthophores per mm^2^) in light or dark stripe regions of wild type and mutants. Values are presented as mean± standard deviation. Asterisk indicate the statistical significance compared to the cell density in wild-type light stripe using Student's *t*-test. Wild-type compact (WT-C) in light stripe (LS): 349.72 cells/mm^2^ ±33.67, Wild-type stellate (WT-S) in dark stripe (DS): 139.82 cells/mm^2^±19.16 (*P*<0.0001); homozygous *shady* light stripe (*shady* -LS): 116.26 cells/mm^2^±14.55 (*P*<0.0001), homozygous *nacre* light stripe (*nacre* -LS): 369.54 cells/mm^2^±24.30, homozygous *nacre* dark stripe (*nacre* -DS): 229 cells/mm^2^ ±18, heterozygous, *leo*^tK3^ light stripe (*leo^tK3/+^* - LS): 168.03 cells/mm^2^±21.06 (*P*<0.0001), homozygous *leo^tK3^* light stripe (*leo^tK3/tK3^* - LS): 148.71 cells/mm^2^±12.28 (*P*<0.0001). *n*=10 adult (90 dpf) fish per genotype, except for *nacre* dark stripe, *n*=8.

In adult zebrafish xanthophores are present in light stripes and dark stripes. In the dark stripes they cover the melanophores at relatively low density, display a stellate shape and faint coloration; and in the light stripes xanthophore density is much higher, the cells are more compact and more intensely pigmented (Fig. 1A,B1-B5) (Hirata et al., 2003; Mahalwar et al., 2014). To investigate whether these differences depend upon the presence of iridophores and melanophores, and their interactions with xanthophores, we analyzed xanthophore distribution in *shady*, which lack most iridophores (Fig. 1C), in *nacre*, which lack melanophores (Fig. 1D), and in *leo^tK3^*, in which gap junction-mediated cell-cell interactions among xanthophores and melanophores are abolished in a dose-dependent manner (*leo^tK3^* homozygote in Fig. 1E; *leo^tK3^* heterozygote in Fig. 1F). Fig. 2 shows xanthophore distribution and morphology in the skin of adult zebrafish in these mutants and in wild type (Fig. 2A1-E4, quantification in Fig. 2F; see Materials and Methods for labeling and counting of xanthophores). Dense iridophores that show an epithelial-like organization are present in the light-stripe regions of wild-type fish, in *nac* mutants and in homozygous *leo^tK3/tK3^* mutants, as shown by membrane localized Tjp1A (Fig. S1) (Fadeev et al., 2015).

As compared to the xanthophore density in the light-stripe regions of wild type (≈350±34 cell/mm^2^; *n*=10 animals), we observe very low densities of xanthophores in *shady* (≈116±15 cells/mm^2^; *n*=10 animals), and in *leo^tK3^* mutants (≈168±21 cells/mm^2^; *n*=10 animals) in heterozygotes, and (≈149±12 cells/mm^2^; *n*=10 animals) in homozygotes. In *nacre* mutants, where no melanophores are present and dense iridophores form only a rudimentary first light stripe with irregular borders towards loose iridophores characteristic of dark stripes (Fig. 1D) (Frohnhöfer et al., 2013), we find that xanthophores covering the dense epithelial-like sheet of iridophores show roughly the same density (≈370±24 cells/mm^2^; *n*=10 animals) as in the light stripes of wild-type fish. Thus iridophores, but not melanophores, are necessary for the high density of xanthophores in the light-stripe regions. In the regions where loose iridophores are present in *nac* mutants, corresponding to dark-stripe regions in wild type, the density of xanthophores is significantly lower (≈229±18 cells/mm^2^; *n*=8 animals), but still higher than in wild-type dark stripes. This suggests that the reduction in the density of xanthophores in the dark stripes is dependent on melanophores. Strikingly, we find a net of xanthophores of uniform low density covering the epithelial-like dense sheet of iridophores in *leo^tK3^* mutants. This low density of xanthophores, comparable to the density observed in *shd* mutants, indicates a communication defect between xanthophores and iridophores in *leo^tK3^*.

### Xanthophore organization depends upon the presence of epithelial-like dense iridophores and functional gap junctions

**Fig. 3.**
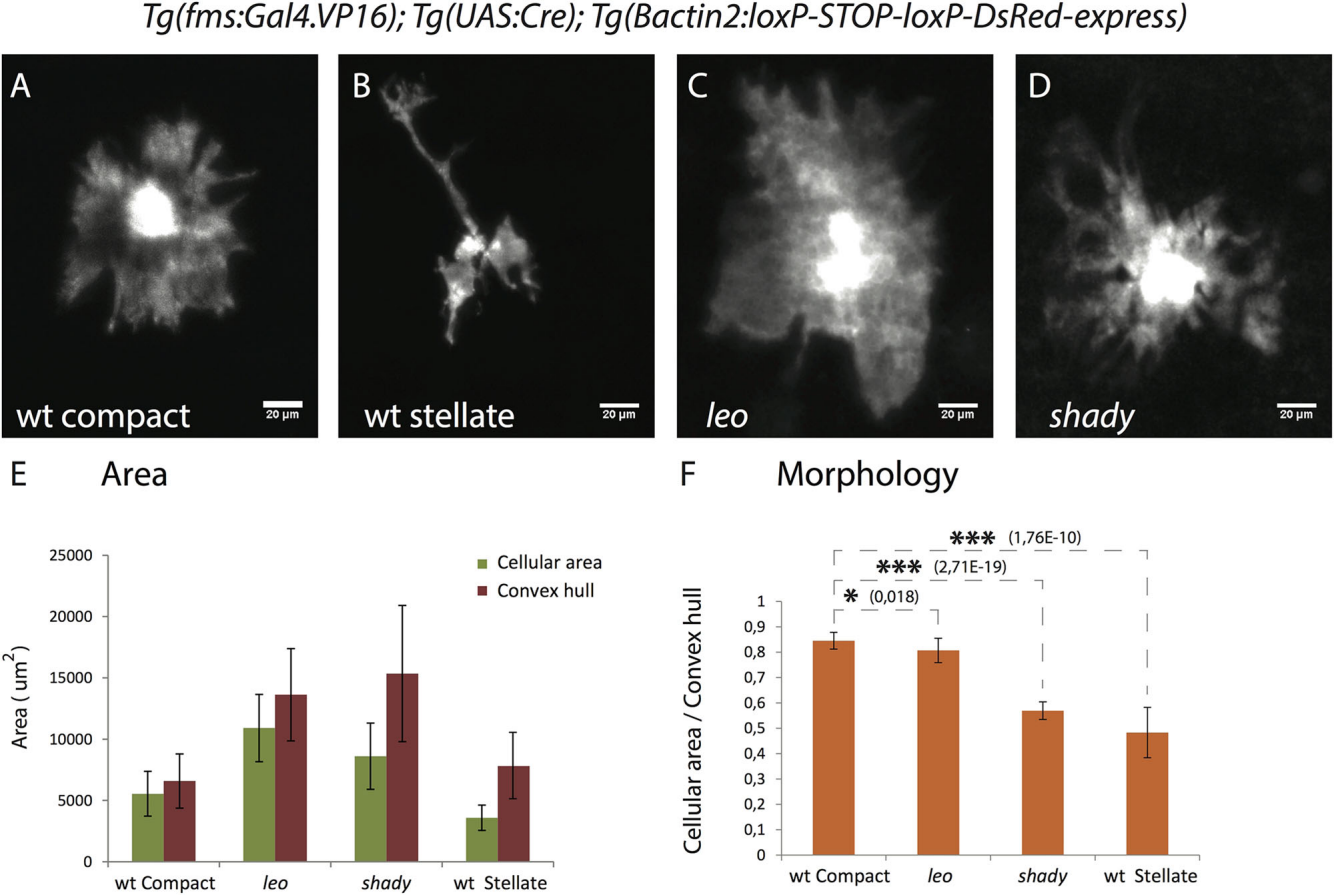
**Xanthophore cell morphologies.** (A-D) High resolution images of xanthophores in wild type and mutants showing the different morphologies. (A) Wild-type compact xanthophore, (B) wild-type stellate xanthophore, (C) *leo^tK3/tK3^* xanthophore and (D) *shady* xanthophore, scale bar: 20 µm. (E) Graph depicting the cell areas (green bars) and the areas of a simple polygon covering the cells (convex hull, red bars) for individual xanthophores in different genotypes (*n*=15 adult (90 dpf) fish per genotype). (F) Graph showing the ratios of cellular areas to convex hull areas as a measure to quantify the differences in cell morphology between wild type, *leo^tK3/tK3^* and *shady* mutants. The ratio for compact xanthophores in wild type is significantly higher than for wild-type stellate xanthophores (*P*<0.0001), xanthophores in *leo^tK3/tK3^* (*P*<0.01) and *shady* (*P*<0.0001) mutants. Asterisks indicate the statistical significance using Student's *t*-test and error bars indicate standard deviations.

The analysis of xanthophore shape and distribution using cell type-specific markers further revealed the role of cell-cell interactions in xanthophore organization (Fig. 2A1-E4; Fig. 3). In wild-type animals, we observed a net of compact and densely packed xanthophores in the light stripe areas (arrowheads in Fig. 2A3-A4), and loose cells with a stellate appearance in the dark stripes (arrow in Fig. 2A3; Fig. S2A). In *shady* mutants, in the absence of iridophores no compact xanthophores are detectable in the light stripes, the cells display a branched morphology with thin cellular projections (Fig. 2B1-B4; arrows in Fig. 2B3-B4). In *nacre*, in the absences of melanophores xanthophores are compact in the regions with dense iridophores (Fig. 2C1-C4; arrowheads in Fig. 2C3-C4). However, in the regions devoid of dense iridophores, the xanthophores do not acquire a compact shape, they appear star-like and are loosely packed (arrows in Fig. 2C3-C4). Strikingly, we found a uniform distribution of xanthophores, albeit at low density, in the trunk along the dorso-ventral axis in homozygous *leo^tK3^* mutants (Fig. 2D1-D4; arrowheads in Fig. 2D3-D4). To further investigate the consequences of non-functional gap junction channels on xanthophore behavior *in vivo*, we imaged labeled xanthophores in wild-type and *leo^tK3^* mutant fish at 15 mm standard length (SL), the stage when mutants start to differ phenotypically from wild type. The uniform distribution of xanthophores was visible in *leo^tK3^* mutants even at this stage, whereas wild-type animals already displayed a higher density of xanthophores in the light stripes as compared to the dark stripes (Fig. S2B). In heterozygous *leo^tK3^* mutants, which produce melanophore spots, the distribution of xanthophores is different between light areas and dark spots (Fig. 2E1-E4). These data indicate that gap junction-dependent cellular interactions with the other two types of chromatophores are necessary for the acquisition of the appropriate size and shape of xanthophores in the skin of zebrafish.

### Xanthophore morphology depends on iridophores

Xanthophores in *leo^tK3^* mutants appear to be larger than in wild type and show a different morphology (Fig. S2). To quantify these differences, we measured the actual cellular area and the area of a simple polygon covering the cell (convex hull) for individual xanthophores from wild type and mutants (see Materials and Methods for the measurement of cell area). In wild type, compact xanthophores of the light stripe (Fig. 3A) and stellate xanthophores of the dark stripes (Fig. 3B) show distinct morphologies, reflected in the different ratios of cellular area to convex hull (Fig. 3E-F). Xanthophores in homozygous *leo^tK3^* mutants show morphology that is neither identical to compact xanthophores of the light stripes nor to stellate cells of the dark stripes in wild type (Fig. 3C; quantification in Fig. 3E-F). We find that the area covered by the xanthophores in *leo^tK3^* mutants is larger than for compact light stripe xanthophores in wild type; however, the ratio of cellular area to convex hull is marginally lower, indicating that the cell morphology is slightly less compact. In the absence of iridophores, in *shady* mutants, xanthophores display an intermediate phenotype, but they are clearly more branched than in *leo^tK3^* mutants (Fig. 3D; quantification in Fig. 3E-F).

**Fig. 4.**
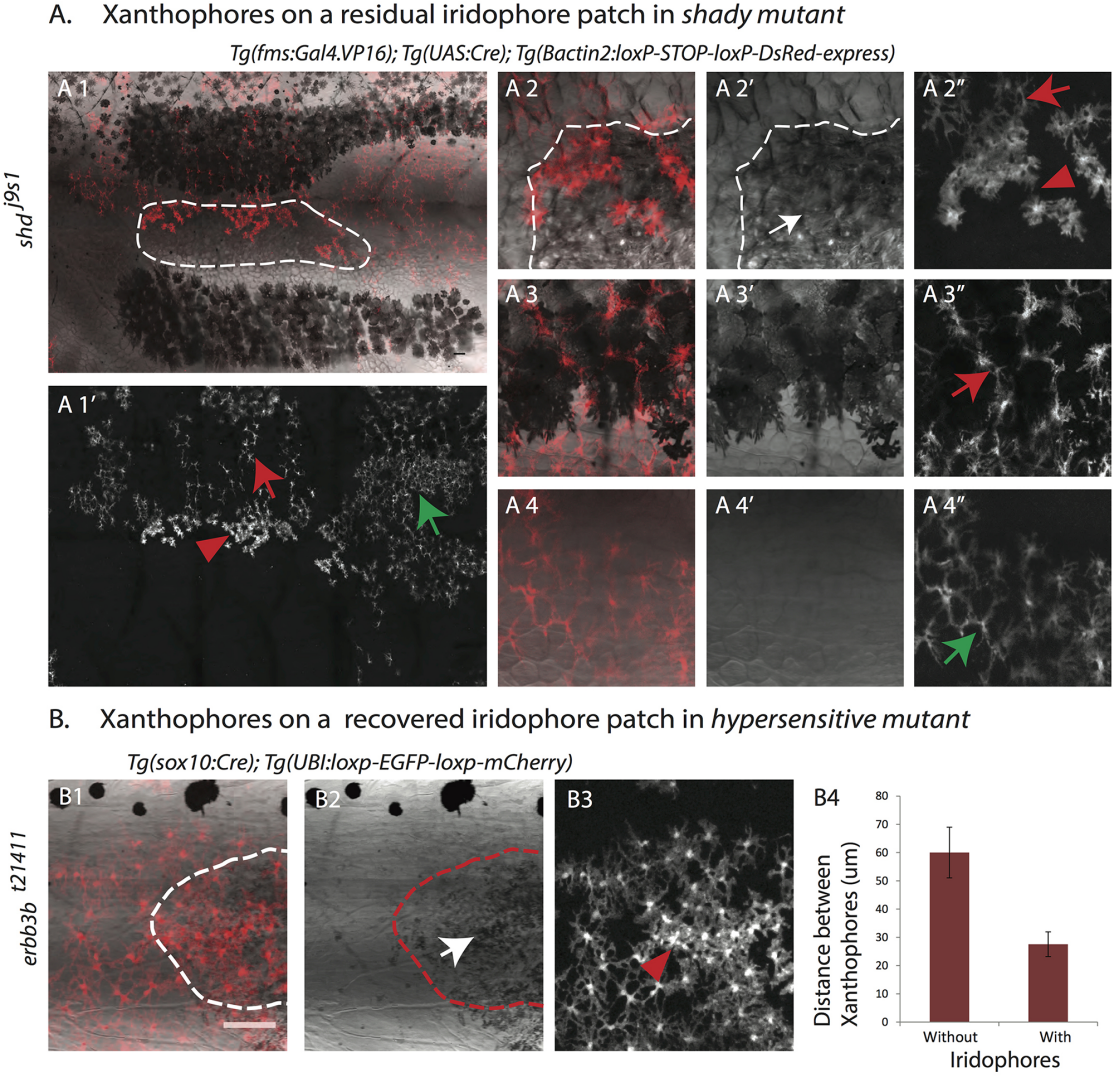
**Iridophores lead to a higher density and change in the organization of xanthophores.** (A) Adult *shady* mutant with DsRed-positive xanthophore patches labeled with *Tg*(*fms:Gal4.VP16*); *Tg*(*UAS:Cre*); *Tg*(*βactin2:loxP-STOP-loxP-DsRed-express*). Xanthophores show a compact organization and higher density (red arrowhead in A1′ and A2″) on top of escaper iridophores (outlined in A1 and white arrow in A2′). They are stellate and loosely packed on top of the dark stripe melanophores (red arrow in A1′ and A3″). (A4-4″) Magnified image of the first light-stripe region shows the stellate shape of xanthophores in the absence of the other two pigment cell types (green arrow in A1′ and A4″). (B) *erbb3^bt21411^* metamorphic fish showing a patch of missing iridophores and patchy clones of mCherry-positive xanthophores labeled with *Tg*(*sox10:Cre*); *Tg*(*UBI:loxp-EGFP-loxp-mCherry*). The recovery of iridophores (outlined in B1 and B2, white arrow in B2) leads to denser and more compact xanthophores (red arrowhead in B3); in the absence of iridophores xanthophores remain at lower density. Scale bar: 100 µm. (B4) Graph showing the distances (in µm) between the centers of neighboring xanthophores in the absence or presence of iridophores (*n*=15 pairs).

To confirm the role of dense iridophores in leading to a higher density and more compact organization of xanthophores, we imaged labeled xanthophores in *shady* and *rose* (Parichy et al., 2000a) mutants. They both lack iridophores, but sometimes produce random small patches of dense ‘escaper’ iridophores (Frohnhöfer et al., 2013). Here we observe a higher density of xanthophores associated with these patches of dense iridophores as compared to the areas outside, where no iridophores are present (Fig. 4A; Fig. S3). A similar situation is found in *erbb3b* mutants (aka *hypersensitive*, *hps* or *picasso*) (Budi et al., 2008). Due to a partial absence of dorsal root ganglia and the associated melanophore and iridophore stem cells, large portions of the body in *erbb3b* mutants are devoid of iridophores and melanophores. *erbb3b^t21411^* is a weak allele, which leads to variable missing patches of iridophores and melanophores (Dooley et al., 2013). However, xanthophores are unaffected in these mutants and are present in the regions lacking melanophores and iridophores. This allowed us to study the density and shapes of xanthophores in the absence of the other two pigment cell types. Consistent with our findings in *shady* and *nacre* mutants, we observed a low density of xanthophores with thin cellular projections in the patches devoid of melanophores and iridophores in *erbb3b* mutants (Fig. 4B). In these mutants iridophores divide and slowly fill the gaps (Dooley et al., 2013; Walderich et al., 2016), as this happens we also see a change in shape and compactness of xanthophores (Fig. 4B), consistent with an instructive role of dense iridophores in this process. These three independent observations confirm that the close spacing and compact organization of xanthophores depends on the interaction with the epithelial-like sheet of dense iridophores.

Taken together, these results show a direct role of iridophores in the regulation of xanthophore behavior, regarding cell shape and density. In the absence of iridophores, in *shady* mutants, xanthophores stay at low density and do not display compact cell shapes. Melanophores are not involved in the change in cell density or morphology of the light stripe xanthophores, as these processes also happen in the absence of melanophores in *nacre* mutants. This suggests that the changes in density and shape of xanthophores are mediated by their local interactions with the epithelial-like dense iridophores of the light-stripe regions, and not by long-range interactions with the melanophores of the dark-stripe regions.

### Arrival of dense iridophores at the onset of metamorphosis promotes an increase in xanthophore density

**Fig. 5.**
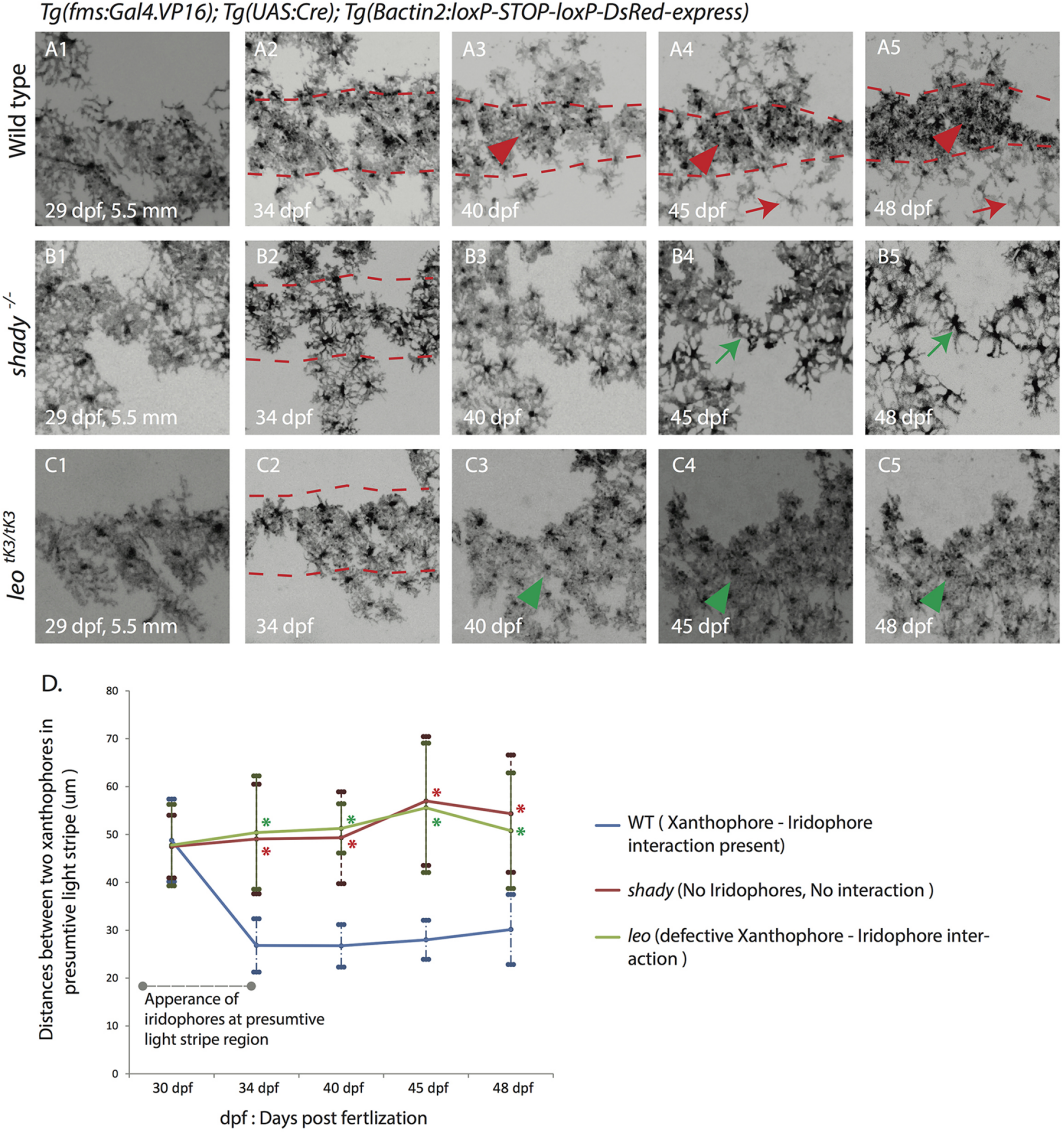
**Development of xanthophore distribution and organization during stripe pattern formation.** Time-lapse images of DsRed-positive xanthophores labeled with *Tg*(*fms:Gal4.VP16*); *Tg*(*UAS:Cre*); *Tg*(*βactin2:loxP-STOP-loxP-DsRed-express*) in wild type and mutants during metamorphosis. (A1-5) Wild-type xanthophores reorganize upon the arrival of metamorphic iridophores and melanophores form a uniform density into two different forms: stellate and less dense (red arrowhead) in the dark-stripe areas (between the red dotted lines), and compact and dense in the light-stripe areas (red arrowheads). (B1-5) Xanthophores in *shady* mutants stay at a uniform density (green arrow) in the dark-stripe areas (between the red dotted lines). (C1-5) In homozygous *leo^tK3^* mutants xanthophores stay at a uniform density (green arrowhead) all over the body of the fish even after the arrival of iridophores and melanophores, dark-stripe areas are shown between red dotted lines. Scale bars: 100 µm. (D) Graph showing the differences in cell densities during development of wild type, *leo^tK3^* and *shady* mutants. The distances (in µm) between the centers of neighboring xanthophores in the prospective light-stripe regions at various developmental time points are depicted (*n*=25 cell pairs from 5 fish per genotype). Error bars indicate standard deviations. Asterisks indicate the statistical significance compared to wild type using Student's *t*-test (all *P* values are *P*<0.0001).

To further investigate the dynamics of the observed xanthophore behaviors, we followed fluorescently labeled xanthophores over several weeks in wild-type, *shady* and *leo^tK3^* mutant fish during metamorphosis (Fig. 5A1-D). We found that in the beginning, before metamorphic iridophores and melanophores appear in the skin, xanthophores cover the body uniformly as a coherent net; there is no difference in the densities and organization of xanthophores in all three cases (Fig. 5A1,B1,C1,D). When iridophores appear along the horizontal myoseptum in the skin of wild-type fish the distances between neighboring xanthophores decrease (Fig. 5D), and consequently the cells become more compact and their density increases, leading to clearly distinct xanthophore populations in the dark and light stripe areas (red arrow and arrowhead in Fig. 5A2-A5; Fig. S4A) (Mahalwar et al., 2014). In *shady* mutants, where no iridophores appear during metamorphosis, distances between neighboring xanthophores do not decrease (Fig. 5D) and the cell density remains low and uniform (green arrow in Fig. 5B2-B5; Fig. S4B). Also in homozygous *leo^tK3^* mutants the density of xanthophores remains low and uniform during the course of metamorphosis (green arrowhead in Fig. 5C1-C5,D; Fig. 4C). This analysis suggests that the cell-cell communication between xanthophores and iridophores in *leo* mutants is affected already during early stages of metamorphosis, before the appearance of melanophores. Therefore we conclude that iridophores and xanthophores interact directly and that gap junctions composed of Cx41.8 and Cx39.4 are involved in these cell-cell communications.

### Multi-level melanophore–xanthophore interactions

**Fig. 6.**
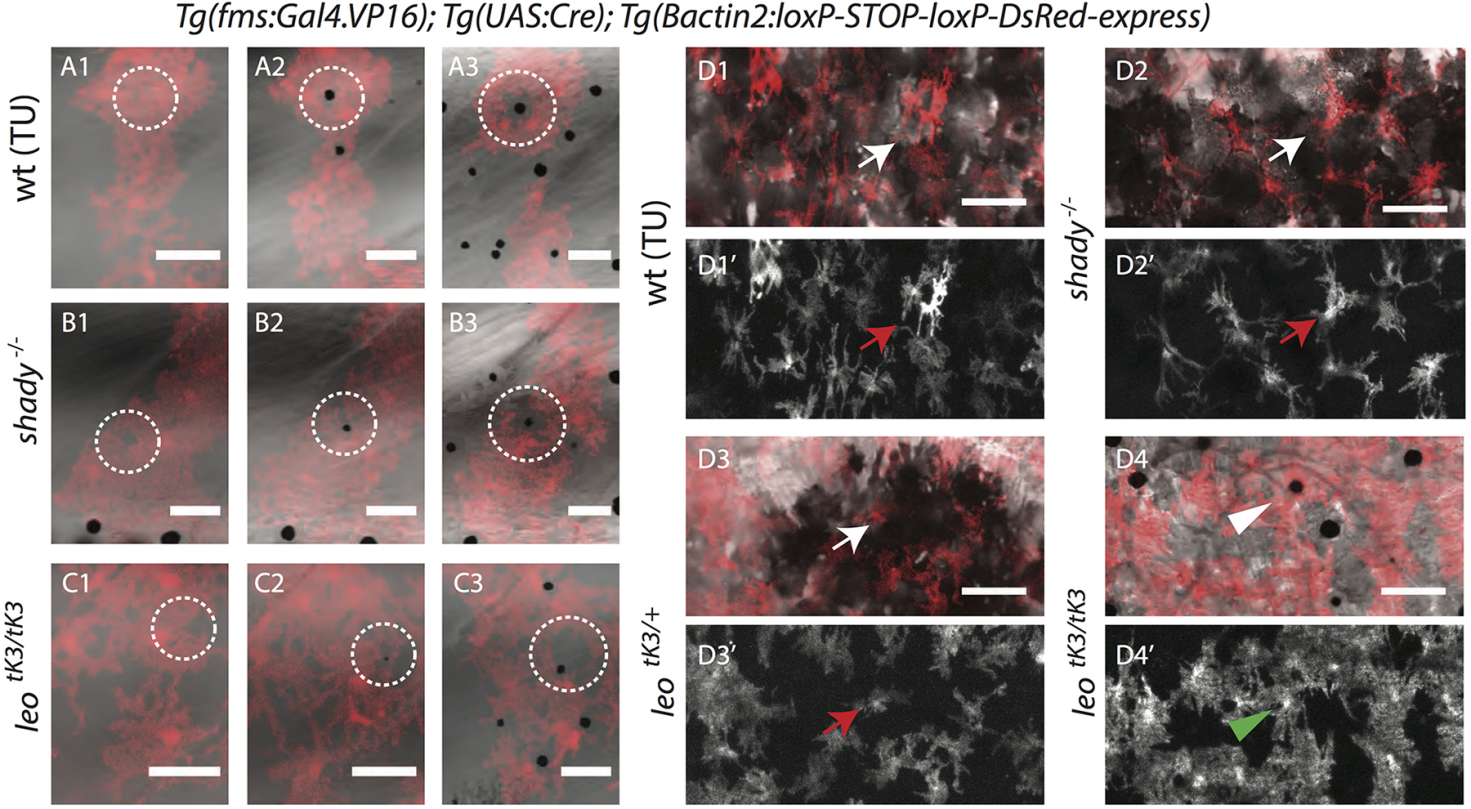
**Xanthophore-melanophore interactions.** (A-C) Time-lapse recordings of melanophores and DsRed-labeled xanthophores during metamorphosis. (A1-3) Wild-type, (B1-3) *shady* and (C1-3) homozygous *leo^tK3^* mutant xanthophores retract their protrusions to give space to the newly arriving melanophores (inside white dotted circles). (D1-1′) Wild-type, (D2-2′) *shady* and (D3-3′) heterozygous *leo^tK3/+^* xanthophores (white and red arrows) reorganize themselves to adopt stellate shapes upon the arrival of more melanophores in the presumptive dark-stripe regions. (D4-4′) In homozygous *leo^tK3/tK3^* mutants xanthophores (green arrowhead, xanthophore above melanophore) do not adopt a stellate shape nor loose morphology. Scale bars: 100 µm.

When melanophores appear in the skin of wild-type fish during metamorphosis, xanthophores in the vicinity retract their cellular protrusions to give way (Fig. 6A1-A3; Fig. S5A). Further, with the appearance of more melanophores in the presumptive dark-stripe regions, xanthophores change their shape and become stellate (Fig. 6D1-D1′) (Mahalwar et al., 2014). To test if these behaviors are dependent on iridophores, which are present in the dark and light-stripe regions, or solely on melanophores in the dark stripes, we observed xanthophores and melanophores in *shady* mutants. We found that xanthophores reorganize their protrusions and become stellate when they encounter melanophores, even in the absence of iridophores (Fig. 6B1-B3,D2-D2′, Fig. S5B). This indicates that direct interactions between melanophores and xanthophores lead to the observed cellular behaviors and that iridophores are not involved in these processes. In homozygous *leo^tK3^* mutants we found that xanthophores also react to newly arriving melanophores by retracting their cellular protrusions, thus making space for the melanophores (Fig. 6C1-C3, Fig. S5C). However, they do not ultimately change their shape to become stellate, like they do in the dark-stripe regions of wild-type fish (Fig. 6D4-D4′). In heterozygous *leo^tK3^* mutants the xanthophores on top of the melanophore spots become stellate, demonstrating that some gap junction intercellular signaling activity is still present in these heterozygotes (Fig. 6D3-D3′). From this analysis, we conclude that melanophores first induce xanthophores to retract their cellular protrusions, followed later by the acquisition of the characteristic stellate morphology of the xanthophores in the dark stripe. Whereas the early aspects of this interaction are *leo-*independent, the acquisition of the final stellate shape does depend upon *leo*-mediated interactions between melanophores and xanthophores, as it does not occur in *leo^tk3^* mutants.

### Gap junctions are cell-autonomously required in xanthophores for their cell shape transition

**Fig. 7.**
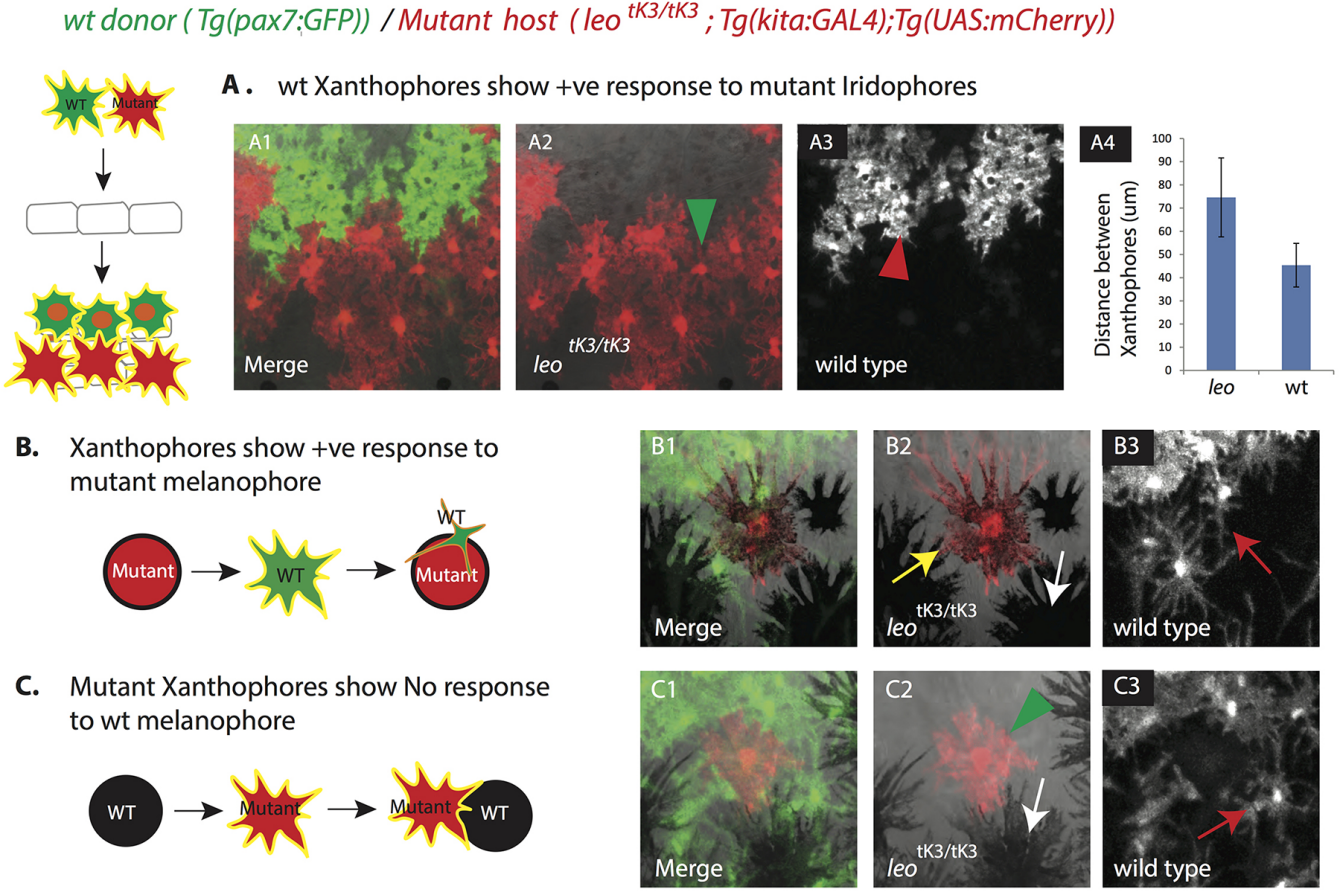
**Transplantation of wild**-**type xanthophores labeled with *Tg*(*pax7:GFP*) into *leo^tK3/tK3^* mutants carrying *Tg*(*kita:GAL4*);*Tg*(*UAS:mCherry*).** (A1) Different density of wild-type (green cells, red arrow head in A3) and mutant xanthophores (red cells, green arrow head in A2) in an adult chimeric animal. (A4) Graph showing the distances (in µm) between the centers of neighboring wild-type and mutant xanthophore pairs in the chimera, error bars indicate standard deviation (*n*=15 pairs). (B) A wild-type xanthophore (labeled with GFP) becomes stellate (red arrow in B3) in response to a mutant melanophore (labeled with mCherry, yellow arrow in B2). White arrow in B2 points to wild-type melanophores. (C) No response and change in shape of mutant xanthophores (labeled with mCherry, green arrowhead in C2) in response to wild-type melanophores (no fluorescent label, white arrow in C2). Red arrow in C3 points to wild-type xanthophores (labeled with GFP) showing wild-type morphology in response to wild-type melanophores.

When we transplanted *Tg*(*pax7:GFP*)-labeled wild-type xanthophores into homozygous *leo^tK3^* mutant hosts carrying *Tg*(*kita:GAL4,UAS:mCherry*), which labels xanthophores and melanophores, we found a clear difference in the density and shape between wild-type and mutant xanthophores in the resulting chimeric animals. The wild-type cells are compact and more densely organized than the mutant cells (Fig. 7A). Further, in dark-stripe regions wild-type xanthophores acquire a stellate shape even in the presence of mutant melanophores (Fig. 7B). Conversely, mutant xanthophores do not respond to the presence of wild-type melanophores; however, wild-type xanthophores do respond and show stellate shapes (Fig. 7C). This shows that gap junctions are autonomously required in xanthophores for the dense and compact organization in the light stripes and the stellate shapes in the dark stripes.

## DISCUSSION

In this study we analyzed the cell-cell interactions during pigment pattern formation in zebrafish from a xanthophore perspective. The outcome of cell-cell interactions depends upon the chromatophore types involved. We observe that heterotypic (xanthophore-iridophore and xanthophore-melanophore) interactions regulate differential density and shape of xanthophores. It was recently shown that homotypic (xanthophore-xanthophore) interactions regulate the xanthophore coverage in the skin (Walderich et al., 2016). Taken together, we conclude that a combination of homotypic and heterotypic interactions regulate precise patterning of xanthophores during color pattern formation.

We found that heterotypic interactions with epithelial-like dense iridophores are required for xanthophores to increase their compactness in light-stripe regions. In the absence of iridophores, in *shady* mutants, xanthophore density stays low and does not increase during metamorphosis. When iridophores gradually recover in *erbb3b* mutants and start to fill in gaps previously devoid of iridophores, xanthophore density also increases. This xanthophore behavior does not depend on the presence of melanophores, as it also occurs in *nacre* mutants. Previously it was reported that iridophores act on xanthophores via Csf1, an extracellular ligand expressed in iridophores that promotes xanthophore development (Patterson and Parichy, 2013). However, the interaction we find between dense iridophores and xanthophores is likely to be more direct, as it requires functional gap junctions made from Cx41.8 and Cx39.4. In *leo^tK3^* mutants, where these channels are non-functional, xanthophores stay at low density despite the presence of iridophores. Our transplantation experiments show that this requirement is cell-autonomous to xanthophores, which is in agreement with our previous findings that Cx41.8 and Cx39.4 are only required in xanthophores and melanophores, but not in iridophores (Irion et al., 2014a; Maderspacher and Nüsslein-Volhard, 2003). If this heterotypic interaction among iridophores and xanthophores occurs indeed via gap junctions, and not simply via hemi-channels in the xanthophore plasma membrane, a different connexin on the iridophore side must exist. Such a connexin has not yet been identified, however, our hypothesis that it exists might be corroborated by the analysis of *schachbrett* (*sbr*) mutants, where a spotted pattern is produced. It was shown that Tjp1A is affected in this mutant, the protein is specifically expressed in iridophores and known to interact with the C-termini of several connexins (Fadeev et al., 2015). This could provide the link from gap junctions to the cytoplasm in iridophores. Reverse genetic approaches may be useful in identifying novel iridophore-specific gap junction components (Irion et al., 2014b; Hwang et al., 2013a,b).

The interactions between xanthophores and melanophores, which lead to shape changes in the xanthophores, are only partially mediated by the known gap junctions, as in homozygous *leo^tK3^* mutants we still observe that xanthophores clearly sense the appearing melanophores and rearrange their cellular protrusions; however, they do not ultimately change their shape to become stellate. This indicates that there are at least two steps in the interactions between melanophores and xanthophores and that the initial recognition is independent of *leo* and *luc* gap junctions. The signaling molecules that mediate this initial interaction are unknown; however, several molecules such as Kcnj13, an inward rectifying potassium channel (Inaba et al., 2012); spermidine, a polyamine (Frohnhöfer et al., 2016); Tetraspanin3c, a transmembrane scaffolding protein (Inoue et al., 2014); and Notch/Delta signaling (Eom et al., 2015; Hamada et al., 2014) have been identified that regulate cell-cell interactions during color pattern formation. It is possible that some of these molecules mediate gap junction-independent communication between melanophores and xanthophores.

Later steps during the interactions between melanophores and xanthophores are dependent on functional gap junctions as they don't occur in homozygous *leo^tK3^* mutants. However, our transplantation experiments show that wild-type xanthophores can still respond to mutant melanophores, suggesting that on the melanophore side different connexins could be involved in the generation of heterotypic and heteromeric gap junctions. Due to its amenability to genetic and cell biological investigation pigment pattern formation, the zebrafish is an attractive model system to study the formation and function of gap junctions *in vivo*. It will be interesting to see if other connexins, expressed in iridophores or melanophores, can be identified and how they might affect the gating properties of gap junctions.

In summary, we demonstrate that gap junctions are required in xanthophores for the cell shape transitions in response to other chromatophores. We also show that iridophores are an integral part of the cell-cell interaction network responsible for generating the striped pattern. Our previous study suggested that xanthophores and melanophores together instruct the patterning of iridophores, here we show that iridophores, in turn, are required to organize xanthophores, suggesting that ultimately a feed-back mechanism involving contact-based interactions between all three types of pigment cells is the basis of stripe pattern formation in the body of zebrafish. We suggest that color pattern formation in zebrafish involves a novel mechanism of patterning dependent on cell shape transitions of xanthophores and iridophores. These shape transitions are dependent on local cell-cell interactions mediated by gap junctions.

## MATERIALS AND METHODS

### Zebrafish lines

The following zebrafish lines were used: wild type (WT) (TU strain from the Tübingen zebrafish stock center), *nacre^w2^* (Lister et al., 1999), *shady^j9s1^* (Lopes et al., 2008), *leo^tK3^* (Irion et al., 2014a), *rose^tAN17X^* (Krauss et al., 2014), *hps/erbb3b^t21411^* (Dooley et al., 2013), *Tg*(*fms*:*GAL4*) (Gray et al., 2011), *Tg*(*UAS:Cre*) (Mahalwar et al., 2014), *Tg*(*βactin2:loxP-STOP-loxP-DsRed-express*) (Bertrand et al., 2010), *Tg*(*kita:GAL4,UAS:mCherry*) (Anelli et al., 2009), *Tg*(*sox10:Cre*) (Rodrigues et al., 2012) and *Tg*(*UBI:loxp-gfp-loxp-mcherry*) (Mosimann et al., 2011), *Tg*(*UAS*:*EGFP*-*CAAX*) (Fernandes et al., 2012), *Tg*(*pax7:GFP*) (Alsheimer, 2012).

Zebrafish were raised as described previously (Brand et al., 2002). The staging of metamorphic fish was done as described (Parichy, 2006). All animal experiments were performed in accordance with the rules of the State of Baden-Württemberg, Germany and approved by the Regierungspräsidium Tübingen (Aktenzeichen: 35/9185.46-5 and 35/9185.82-7).

### Different methods of labeling xanthophores

Different transgenic lines labeling xanthophores were used in various combinations. All of them are stable transgenic lines, which were crossed into various mutant backgrounds. They label xanthophores using three different promoters: neural crest-specific, *sox 10*; pigment cell-specific, *kitA*; and xanthophore-specific, *fms*. These promoters have been shown to label xanthophores (Anelli et al., 2009; Gray et al., 2011; Mongera et al., 2013). *Tg*(*sox10:Cre*) was used in combination with *Tg*(*UBI:loxp-EGFP-loxp-mCherry*) for the analysis of xanthophores in the *hps* mutant background. *Tg*(*kita:GAL4*) was used in combination with *Tg*(*UAS:mCherry*) to visualize adult xanthophores in *rse* mutants. *Tg*(*fms:Gal4.VP16*) fish were crossed with the following reporter lines to drive fluorophore expression exclusively in xanthophores: *Tg*(*UAS:EGFP-CAAX*), *Tg*(*UAS:Cre*) and *Tg*(*βactin:loxp-STOP-loxp-DsRed*). This combination of four transgenes labels only xanthophores, and was used for the follow-up studies of clusters as well as individual xanthophores. In most of cases not all xanthophores are fluorescently labeled, due to the variegation/patchiness created by the combination of the GAL4, UAS and responder transgenic lines. Variegated labeling allows us to see individual cells, and follow them during the course of development and distinguish between different morphologies.

### Counting of xanthophores

Xanthophore numbers were counted using the scans with multiple channels: transgenic marker (variegation), auto-fluorescence (no variegation) and bright field. Auto-fluorescence marks all the xanthophores; however, double labeling was performed to confirm the presence of xanthophores. Only regions corresponding to the first light stripe were used for counting in all the mutants. Xanthophore counting was done using the ‘cell counter’ plugin in Fiji (Schindelin et al., 2012). Ten readings from ten fish per genetic background were used to obtain the densities and respective standard deviations.

### Area, distance and density of xanthophores

High resolution images were taken to resolve differences between different morphologies of xanthophores in various backgrounds. To calculate the cellular areas, image thresholds were set using the mean algorithm and the values were calculated using the measure option in ImageJ. To obtain a quantitative measure for the different cell morphologies, the convex hull area value of the corresponding cell was also calculated. The final values shown in Fig. 1 were calculated as the ratios between the total cellular areas and the areas of the convex hull. 15 individual cells per genetic background were used to calculate the areas and standard deviation values in Fig. 1. Distances between the centers of neighboring xanthophores were measured using ImageJ.

### Image acquisition and processing

Repeated imaging of zebrafish was performed as described in Singh et al. (2014). Images were acquired on a Zeiss LSM 780 NLO confocal microscope. Fiji (Schindelin et al., 2012) and Adobe Illustrator were used for image processing and analysis. Maximum intensity projections of confocal scans of the fluorescent samples were uniformly adjusted for brightness and contrast. Scans of the bright field were stacked using the ‘stack focuser’ plugin and tile scans.

### Blastomere transplantations

Chimeric animals were generated by transplantation of few cells from wild-type embryos carrying *Tg*(*pax7:GFP*) into *leo^tK3/tk3^* mutant embryos carrying *Tg*(*kita:GAL4,UAS:mCherry*) at blastula stage (Kane and Kishimoto, 2002).

### Immunohistochemistry

Antibody stainings were performed as described previously (Fadeev et al., 2015). anti-Tjp1aC was used 1:100, as secondary antibody Alexa Fluor 488 goat anti-mouse (Invitrogen/Molecular Probes, A11008) was used 1:400.
